# Supplementary material for: Active pulmonary tuberculosis and coronavirus disease 2019: A systematic review and meta-analysis
Source: PLoS One. 2021 Oct 21;16(10):e0259006. doi: 10.1371/journal.pone.0259006 (PMC8530351; doi:10.1371/journal.pone.0259006)
Supplement: S1 Table — (PDF) [file pone.0259006.s001.pdf]

S1 Table. Scoring of the Newcastle-Ottawa Quality Assessment Scale adapted for meta-analysis.

Study author, year	Selection				Comparability		Outcome			Total score (maximum 9)
	Exposed cohort representative	Selection of non-exposed cohort	Ascertainment of exposure	Outcome not present at start of study	Study controls for age	Study controls for gender or other factors	Outcome assessment adequate	Long enough follow up	Adequacy of follow up	
Al Kuwari HM, 2020	Yes	Yes	Yes	No	No	No	Yes	Yes	Yes	6
Boulle A, 2020	Yes	Yes	Yes	No	Yes	Yes	Yes	Yes	Yes	8
Chen T, 2020	Yes	Yes	Yes	No	No	No	Yes	Yes	Yes	6
Dai M, 2020	Yes	Yes	No	No	No	No	Yes	Yes	Yes	5
Du RH, 2020	Yes	Yes	No	Yes	No	No	Yes	Yes	Yes	6
Gupta N, 2020	Yes	Yes	No	Yes	No	No	Yes	No	Yes	5
Ibrahim OR, 2020	Yes	Yes	Yes	No	No	No	Yes	Yes	Yes	6
Lee SG, 2020	Yes	Yes	Yes	No	Yes	No	Yes	Yes	Yes	7
Li G, 2020	Yes	Yes	No	No	Yes	Yes	Yes	Yes	Yes	7
Li X, 2020	Yes	Yes	Yes	No	No	No	Yes	Yes	Yes	6
Liu J, 2020	Yes	Yes	Yes	No	No	No	Yes	Yes	Yes	6
Liu S, 2020	Yes	Yes	No	No	No	No	Yes	Yes	Yes	5
Ma Y, 2020	Yes	Yes	Yes	No	No	No	Yes	Yes	Yes	6
Maciel EL, 2020	Yes	Yes	No	No	No	No	Yes	Yes	Yes	5
Nachega JB, 2020	Yes	Yes	No	No	Yes	No	Yes	Yes	Yes	6
Parker A, 2020	Yes	Yes	No	No	No	No	Yes	Yes	Yes	5
Sun Y, 2020	Yes	Yes	No	No	No	No	Yes	Yes	Yes	5
Sy KTL, 2020	Yes	Yes	Yes	No	Yes	Yes	Yes	Yes	Yes	8
Xiao K, 2020	Yes	Yes	No	No	No	No	Yes	Yes	Yes	5
Yu HH, 2020	Yes	Yes	No	No	Yes	Yes	Yes	Yes	Yes	7
Zeng JH, 2020	Yes	Yes	No	No	No	No	Yes	Yes	Yes	5
Zhang JJ, 2020	Yes	Yes	No	Yes	No	No	Yes	Yes	Yes	6

Zhang Y, 2020	Yes	Yes	No	No	No	No	Yes	Yes	Yes	5
Abraha HE, 2021	Yes	Yes	No	No	Yes	Yes	Yes	Yes	Yes	7
Dave JA, 2021	Yes	Yes	Yes	No	No	No	Yes	Yes	Yes	6
du Bruyn, 2021	Yes	Yes	Yes	No	No	No	Yes	Yes	Yes	6
Gajbhiye RK, 2021	No	Yes	No	No	No	No	Yes	Yes	Yes	4
Hesse R, 2021	Yes	Yes	Yes	No	No	No	Yes	Yes	Yes	6
Kapoor D, 2021	No	Yes	No	No	No	No	Yes	Yes	Yes	4
Lagrutta L, 2021	Yes	Yes	Yes	No	No	No	Yes	Yes	Yes	6
Li S, 2021	Yes	Yes	No	No	No	No	Yes	No	Yes	4
Lu Y, 2021	Yes	Yes	No	No	No	No	Yes	Yes	Yes	5
Meng M, 2021	Yes	Yes	No	No	No	No	Yes	Yes	Yes	5
Mithal A, 2021	Yes	Yes	No	No	No	No	Yes	Yes	Yes	5
Moolla MS, 2021	Yes	Yes	No	Yes	No	No	Yes	Yes	Yes	6
Song J, 2021	Yes	Yes	Yes	No	No	No	Yes	Yes	Yes	6
van der Zalm MM, 2021	No	Yes	Yes	No	No	No	Yes	Yes	Yes	5
Verma R, 2021	No	Yes	No	No	No	No	Yes	Yes	Yes	4
Yan B, 2021	Yes	Yes	No	No	No	No	Yes	Yes	Yes	5
Yang C, 2021	Yes	Yes	No	No	Yes	No	Yes	Yes	Yes	6
Yitao Z, 2021	Yes	Yes	No	No	No	No	Yes	Yes	Yes	5
Zhang W, 2021	Yes	Yes	No	No	No	No	Yes	Yes	Yes	5
Zheng B, 2021	Yes	Yes	No	No	No	No	Yes	Yes	Yes	5
