# Supplementary material for: Active pulmonary tuberculosis and coronavirus disease 2019: A systematic review and meta-analysis
Source: PLoS One. 2021 Oct 21;16(10):e0259006. doi: 10.1371/journal.pone.0259006 (PMC8530351; doi:10.1371/journal.pone.0259006)

S1 Fig. Baujat's plot to identify studies that potentially contribute to heterogeneity, and influence the overall results, more than the others in the meta-analysis of studies reporting proportion of COVID-19 patients having active tuberculosis. Each circle represents an individual study, and those contributing more to heterogeneity are marked in red.

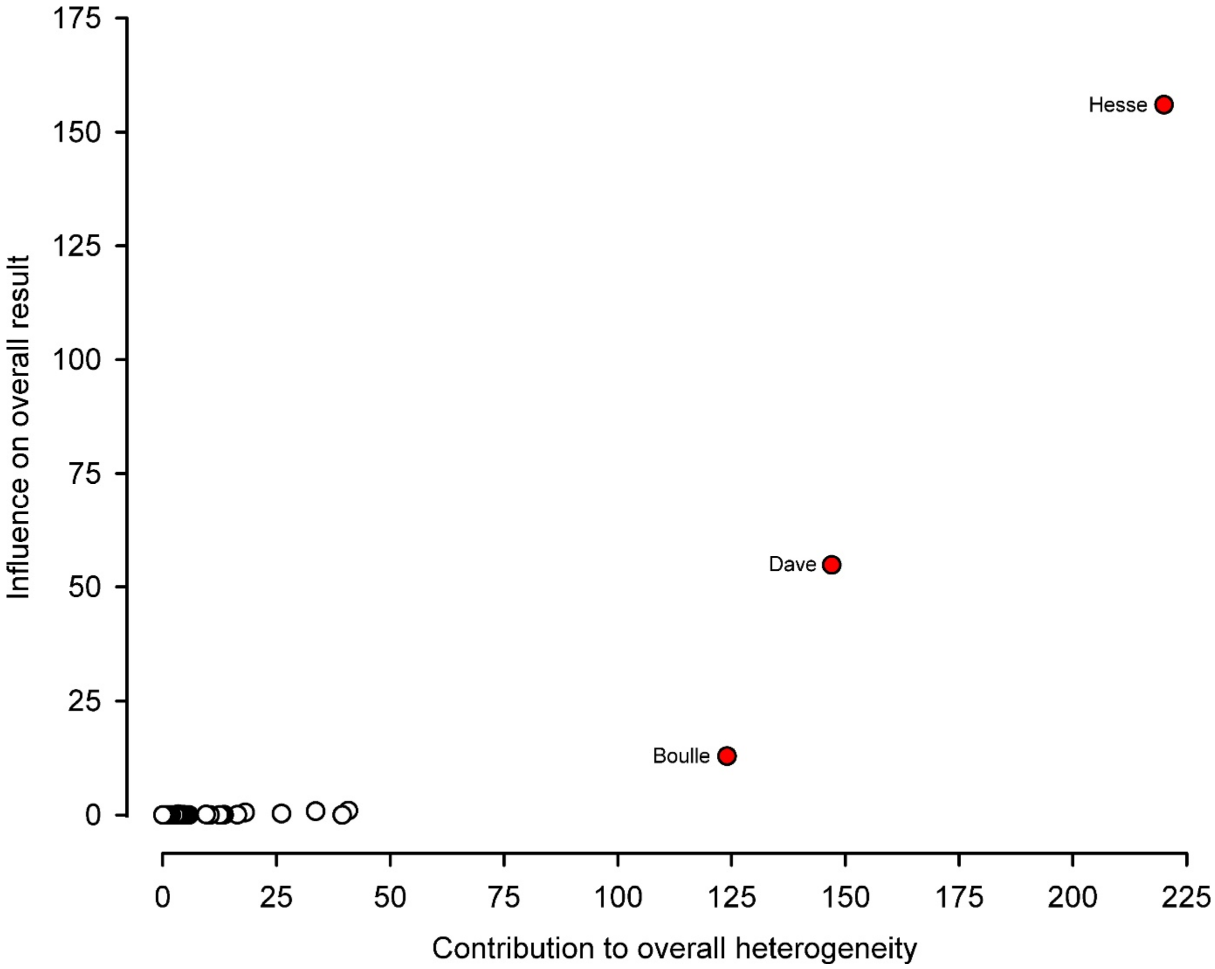

Supplement: S1 Fig — (PDF) [file pone.0259006.s002.pdf]
