# Supplementary material for: Active pulmonary tuberculosis and coronavirus disease 2019: A systematic review and meta-analysis
Source: PLoS One. 2021 Oct 21;16(10):e0259006. doi: 10.1371/journal.pone.0259006 (PMC8530351; doi:10.1371/journal.pone.0259006)

S2 Fig. Influence analysis of 43 publications reporting on proportion of active tuberculosis patients among those having COVID-19. A single study (marked in red) significantly influenced Studentized residuals, Cook's distance, and Difference in Fits (DFFITS).

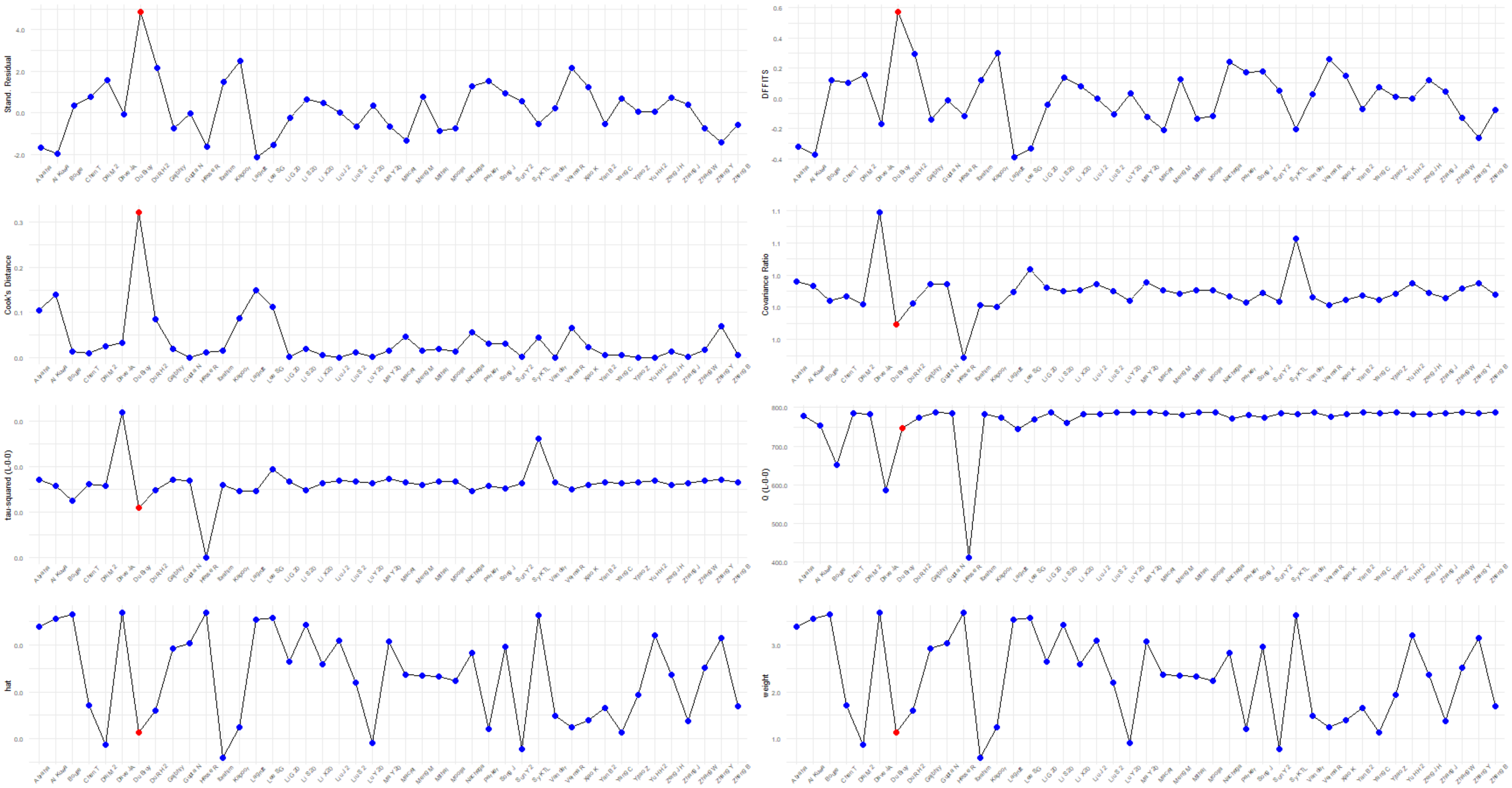

Supplement: S2 Fig — (PDF) [file pone.0259006.s003.pdf]
