# Supplementary material for: Active pulmonary tuberculosis and coronavirus disease 2019: A systematic review and meta-analysis
Source: PLoS One. 2021 Oct 21;16(10):e0259006. doi: 10.1371/journal.pone.0259006 (PMC8530351; doi:10.1371/journal.pone.0259006)

S4 Fig. Sensitivity analysis of the impact of comorbid tuberculosis on COVID-19 outcomes through the leave-one-study-out approach. Individual summary estimates after removing a single study from meta-analysis are depicted by solid squares, and corresponding 95% confidence intervals by horizontal lines. The vertical lines represent the summary estimate with all studies included, and the shaded area represents the corresponding 95% confidence intervals.

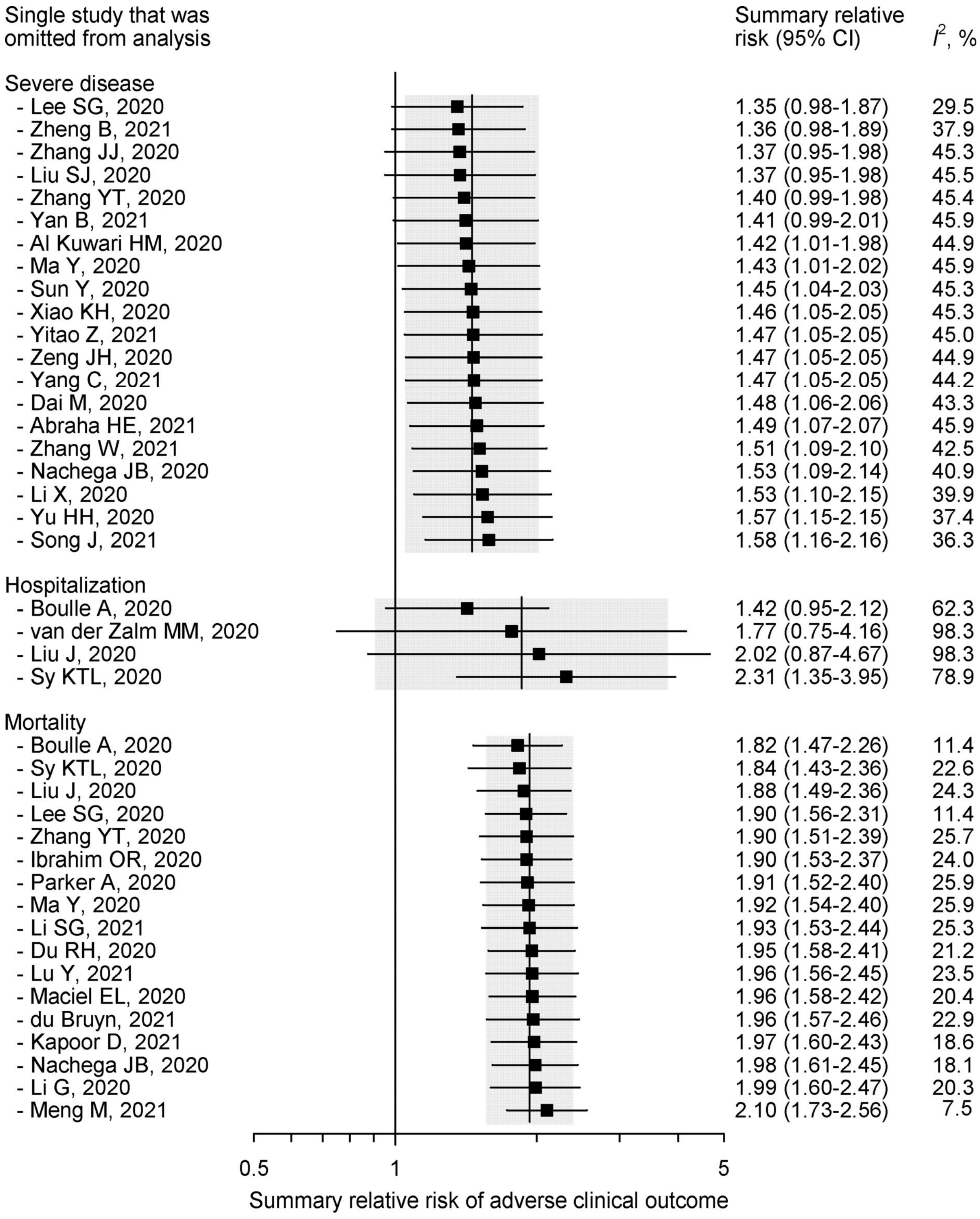

Supplement: S4 Fig — (PDF) [file pone.0259006.s005.pdf]
