# Supplementary material for: Active pulmonary tuberculosis and coronavirus disease 2019: A systematic review and meta-analysis
Source: PLoS One. 2021 Oct 21;16(10):e0259006. doi: 10.1371/journal.pone.0259006 (PMC8530351; doi:10.1371/journal.pone.0259006)

S5 Fig. Contour-enhanced funnel plots of logarithmic risk estimates vs. the standard error for each included study evaluating relative risk of severe COVID-19 (left panel), hospitalization (middle panel) and mortality (right panel), with adjustment using the trim-and-fill approach. Blue circles represent the studies included in each meta-analysis, while red circles represent potentially missing studies imputed after adjusting for funnel plot asymmetry. The vertical red line corresponds to the observed effect size. Differentially shaded areas indicate the level of significance of the effect sizes. No statistically significant assymetry was observed in the analysis for hospitalization or mortality (Egger's test intercept values -0.76 [p=0.823] and -0.89 [p=0.063] respectively). Funnel plot for studies evaluating severe COVID-19 showed significant assymetry (Egger's test intercept value -1.32 [p=0.006]). In plots for severe COVID-19 and mortality data, the potentially missing studies were predominantly located in regions of statistical significance (those with relatively lighter or clear shading). This suggests that the apparent assymetry observed in either analysis is more likely due to reasons other than publication bias.

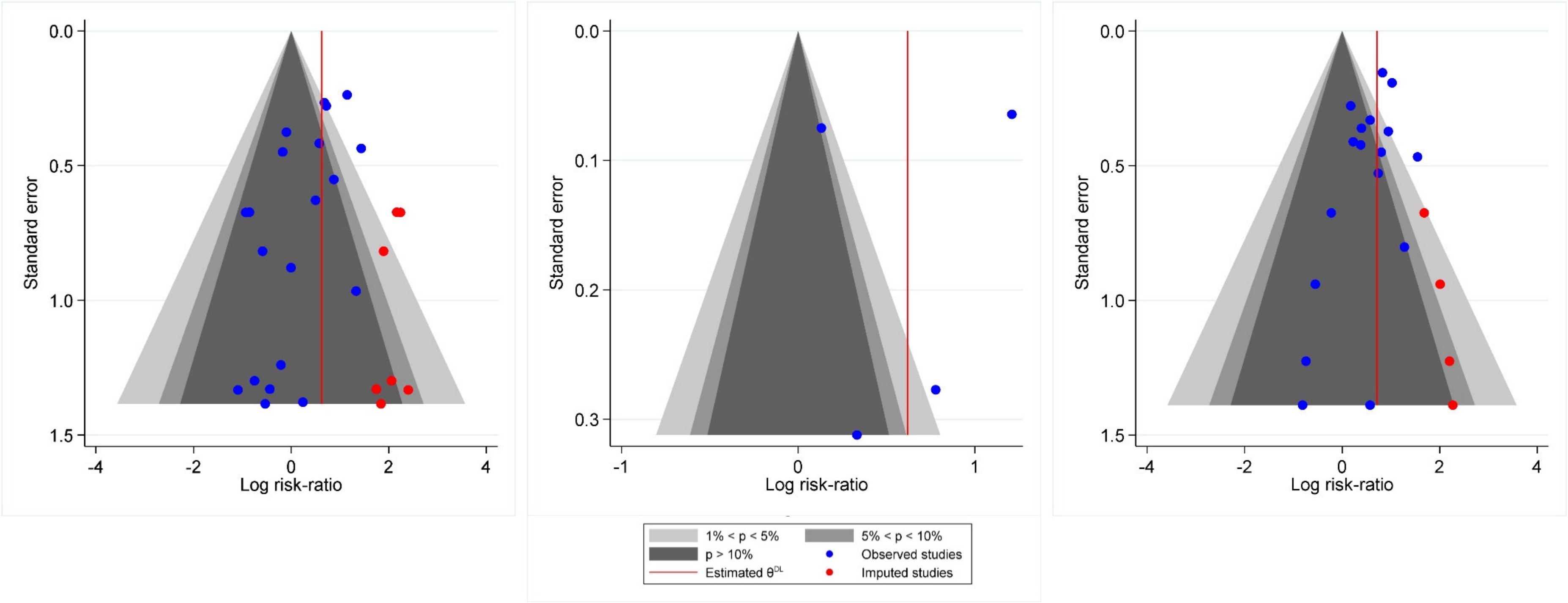

Supplement: S5 Fig — (PDF) [file pone.0259006.s006.pdf]
